# Supplementary material for: Low concordance of short‐term and long‐term selection responses in experimental Drosophila populations
Source: Mol Ecol. 2020 Aug 26;29(18):3466–75. doi: 10.1111/mec.15579 (PMC7540288; doi:10.1111/mec.15579)
Supplement: Supplementary file 1 — Supplementary Material [file MEC-29-3466-s001.zip › mec15579-sup-0001-Supinfo.pdf]

Supplemental Information for

Low concordance of short-term and long-term selection responses in experimental *Drosophila* populations

Anna Maria Langmüller[1,2] and Christian Schötterer[2,*]

[1]Vienna Graduate School of Population Genetics, 1210, Austria
[2]Institut für Populationsgenetik, Vetmeduni Vienna, Veterinärplatz 1, 1210 Wien 1210, Austria
[*]corresponding author: christian.schloetterer@vetmeduni.ac.at

List of Figures

S1 Time resolved candidate window respresentation 3
S2 Haplotype discovery rate 4
S3 Haplotype discovery rate, PCA 5
S4 Similarity measures for candidate SNPs (simulation with linkage) 6
S5 Rank of candidate SNPs over time (simulation with linkage) 7
S6 Similarity measures for candidate SNPs (including fixed SNPs) 8
S7 Number of detected causative SNPs (simulation with linkage) 9
S8 Fraction of shared candidates (including fixed SNPs) 10
S9 Fraction of candidate SNPs fixed at later generations 11
S10 Fraction of shared candidates (simulation with linkage) 12
S11 Early detectable haplotype blocks properties II 13
S12 Early detectable haplotype blocks properties (simulation with linkage) 14
S13 drift can enhance parallelism (simulation without linkage) 15

List of Tables

S1 N_e estimates 16
S2 Replicate-specific autosomal N_e estimates 17
S3 Replicate-specific X N_e estimates 18
S4 Fraction of shared candidate windows 19
S5 Number of candidate windows at each time point 20
S6 Number of candidate SNPs at each time point 21
S7 Number of candidate SNPs at each time point (simulated data) 22
S8 Marginal significant candidate SNPs in blocks with fluctuating HADR 23

Supplementary Material and Methods

Identification of candidate SNPs

SNPs were called applying the following criteria: base quality of 40 in at least one replicate, a read depth between the 2nd and 98th percentile, and the minor allele is supported by at least ten reads. Repeats, transposable elements, SNPs specific to Y-translocated genes (Tobler et al., 2017), and 5-bp regions around indels were excluded from the analysis to increase the robustness of the SNP set (for further details, see Barghi et al. (2019)), resulting in 5,096,200 SNPs on chromosomes X, 2, 3, and 4.

References

Barghi, N., Tobler, R., Nolte, V., Jakšić, A. M., Mallard, F., Otte, K. A., Dolezal, M., Taus, T., Kofler, R., and Schlötterer, C. (2019). Genetic redundancy fuels polygenic adaptation in *Drosophila*. *PLOS Biology*, 17(2):1–31.

Tobler, R., Nolte, V., and Schlötterer, C. (2017). High rate of translocation-based gene birth on the *Drosophila* Y chromosome. *Proceedings of the National Academy of Sciences*, 114(44):11721–11726.

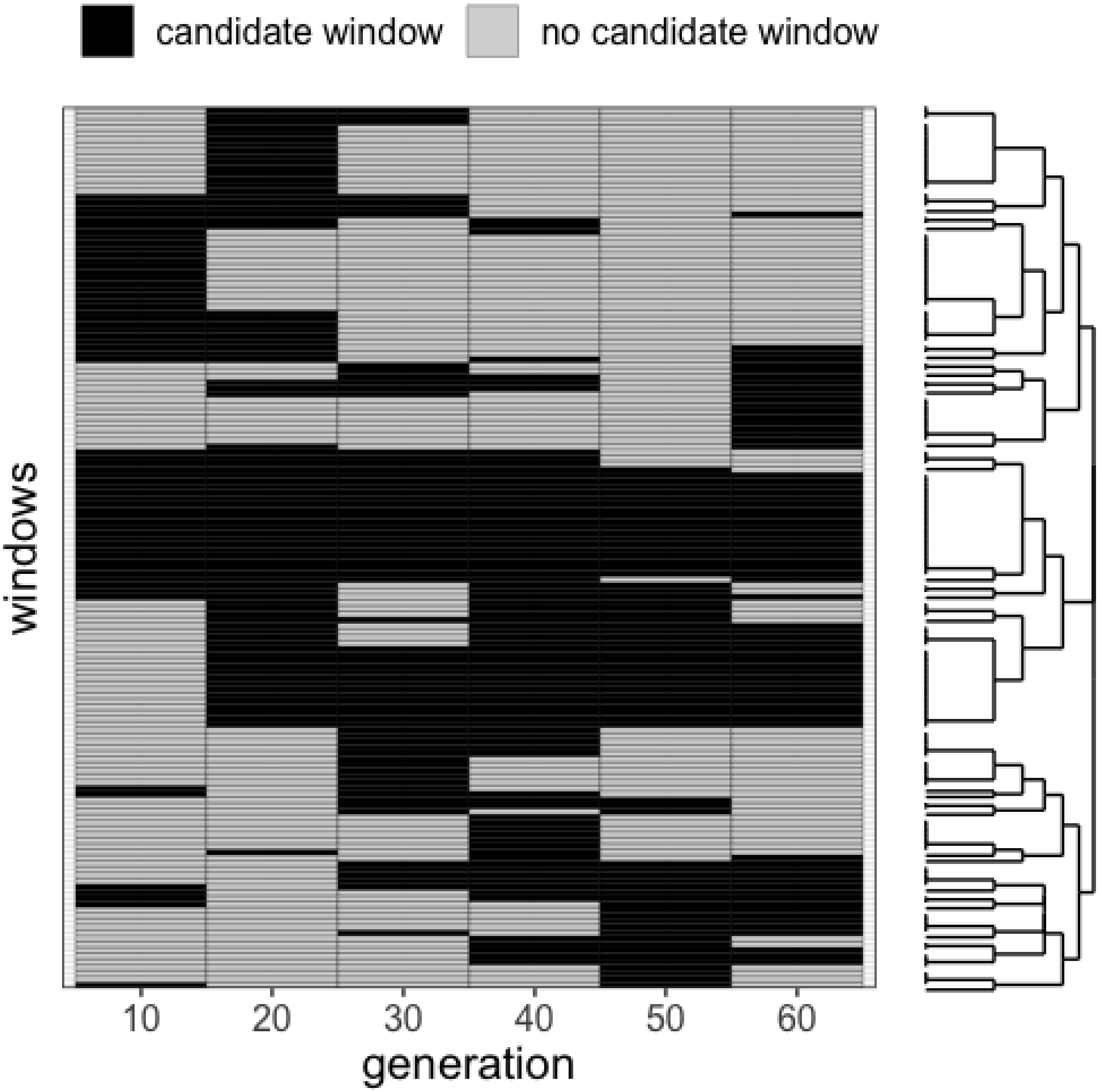

Figure S1: Time resolved representation of all windows that were classified as candidate window for at least one time point: each window is shown in a single row for generation 10 to 60. Windows that are enriched for candidate SNPs at a particular time point are colored black; not enriched windows are colored grey. Windows with similar candidate window patterns are grouped together.

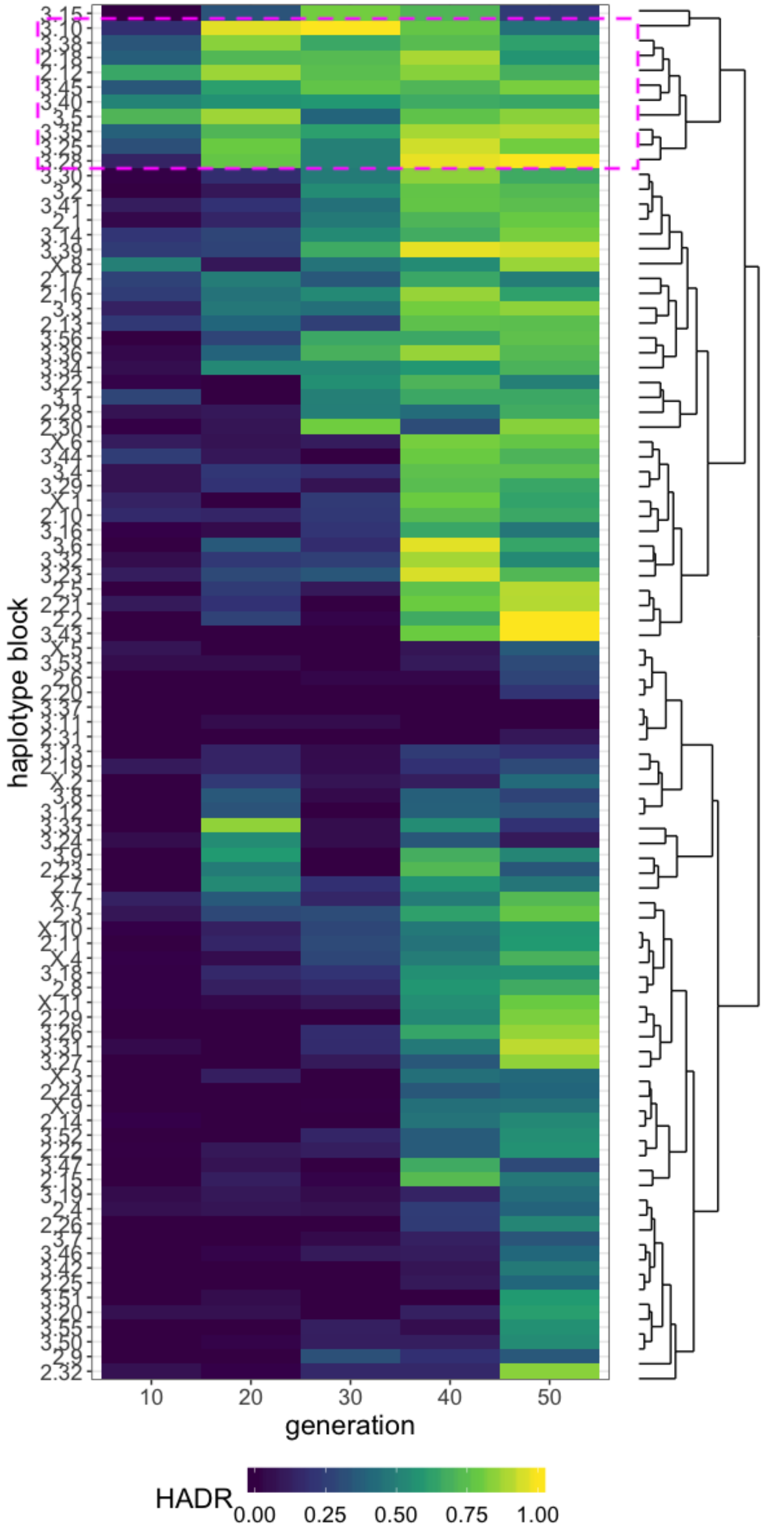

Figure S2: Empirical data: Haplotype discovery rate (HADR) from generation 10 to generation 50. The x-axis shows the five intermediate time points, the y-axis the haplotype blocks determined by Barghi et al. (2019). Haplotype blocks are grouped by similar HADR dynamics over time. Tiles are colored by HADR score. The 10 haplotype blocks of the Early Detected Haplotypes (EDHA) cluster (n=10) are highlighted by a pink frame.

While the HADR of most haplotype blocks increases with time, for nine haplotype blocks the HADR score fluctuated (2.7, 2.15, 2.23, 3.8, 3.9, 3.12, 3.24, 3.33, and 3.47). Because these haplotype blocks are mainly based on marginally significant candidate SNPs (Table S8), they are more sensitive to random fluctuations, partly due to experimental noise, partly due to stochasticity of the simulations used to determine the significance threshold.

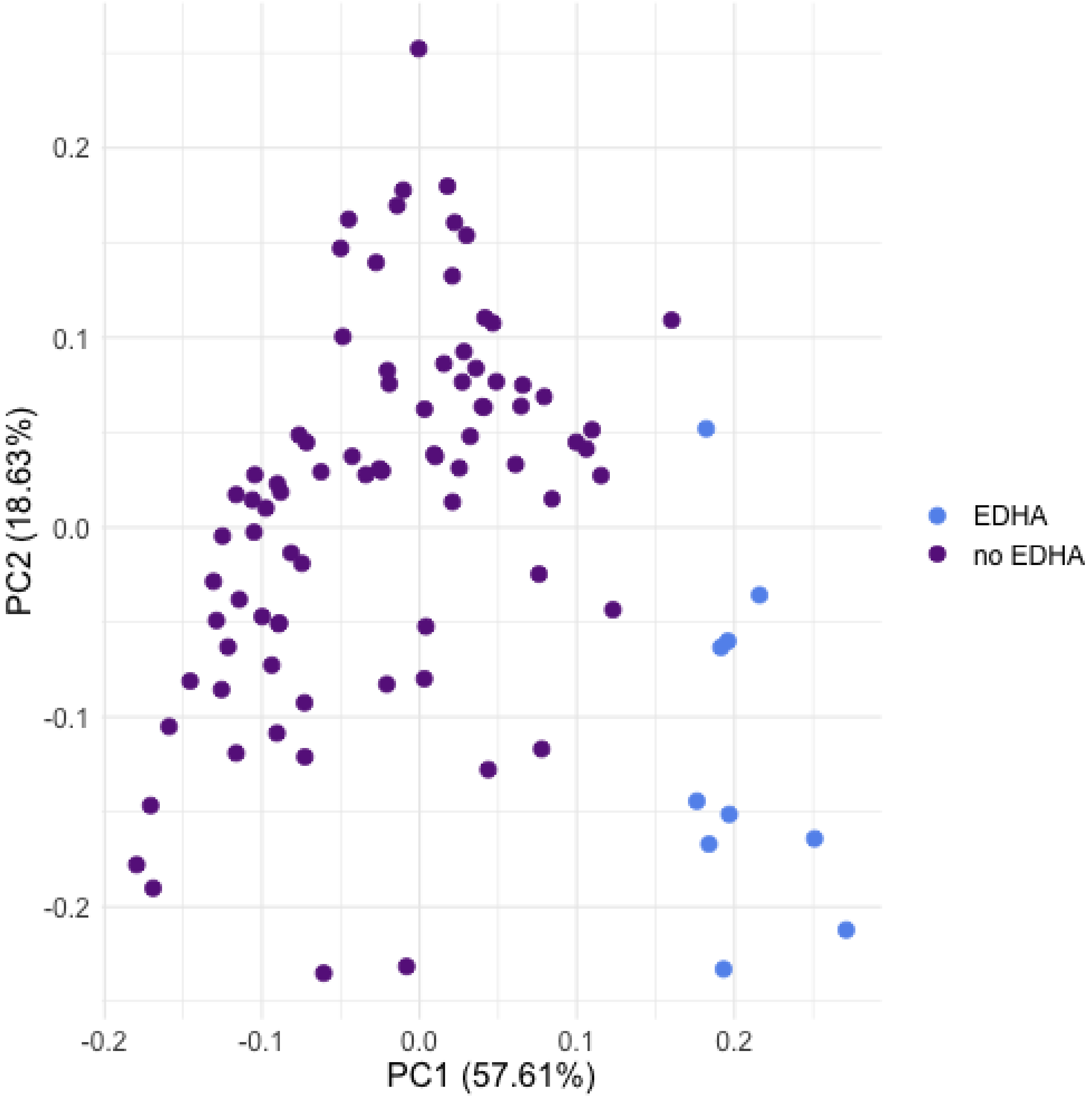

Figure S3: The first two principle components from a PCA based on the haplotype discovery rate (HADR) from generation 10 to generation 50. Each haplotype block is represented by a single dot. Blocks of the early detectable haplotype block (EDHA) cluster are colored in blue, others in purple.

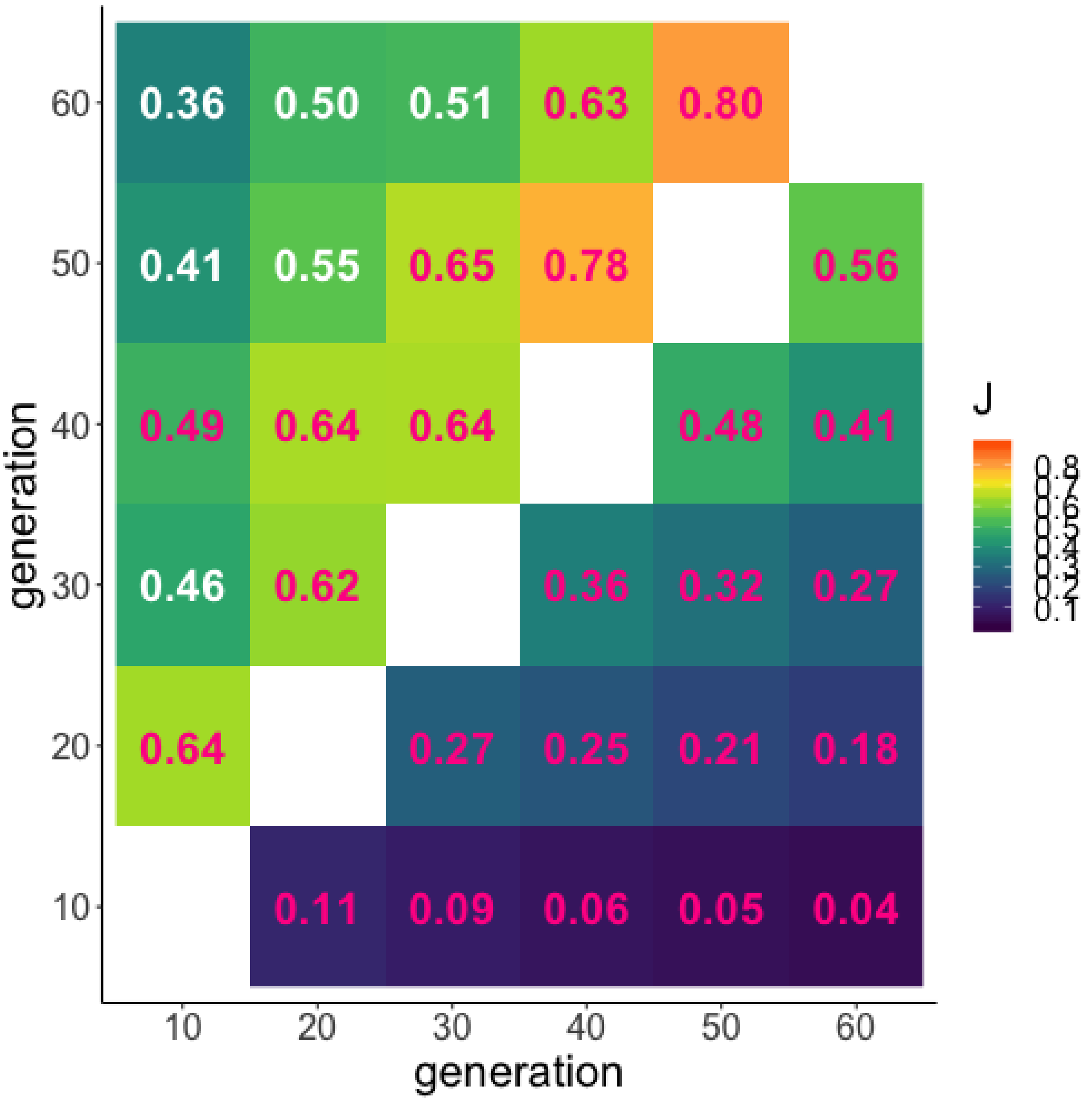

Figure S4: Jaccard index (J) for pairwise comparisons of candidate sets based on a simulated Evolve and Resequence experiment (filtered for SNPs segregating in all generations and time points). The top triangle shows candidate window sets, the bottom triangle candidate SNP sets. Significant similarities (p<0.05 after multiple testing correction, 10 000 bootstraps) are marked in pink.

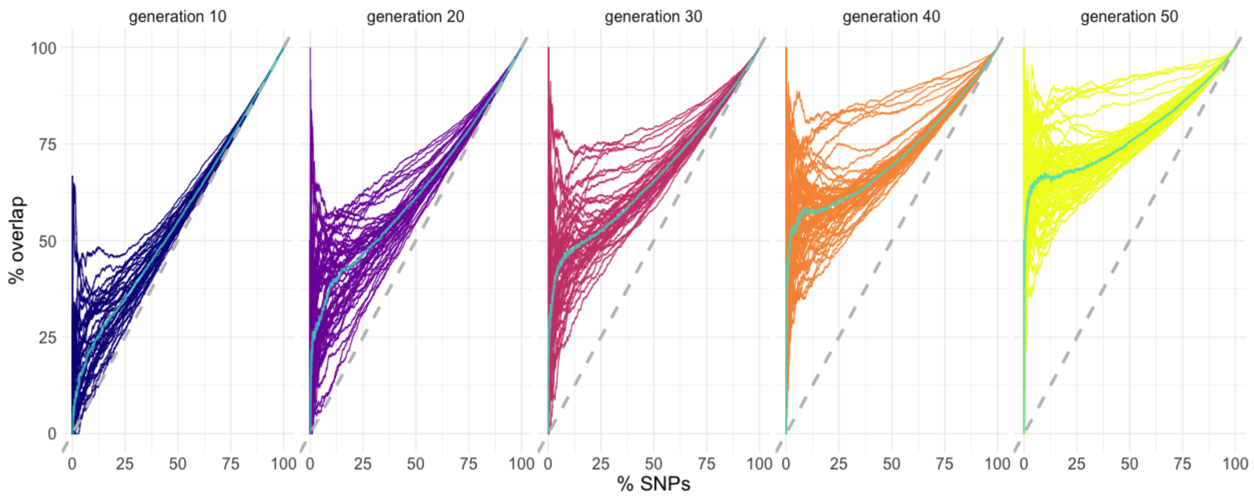

Figure S5: The rank of candidate SNPs becomes more congruent with time in the simulated data. In this ROC-like graph, the ranking of all candidate SNPs in candidate windows is compared. Each panel shows one intermediate time point compared to generation 60. The overlap (in percent) for each candidate window is indicated by a separate line. The median overlap (turquoise line) monotonically increases with experimental duration, demonstrating that the ranking of candidate SNPs is more robust for advanced generations. The grey, dashed lines shows the expected overlap in SNP ranking if every variant at generation 60 is recapitulated in a previous time point.

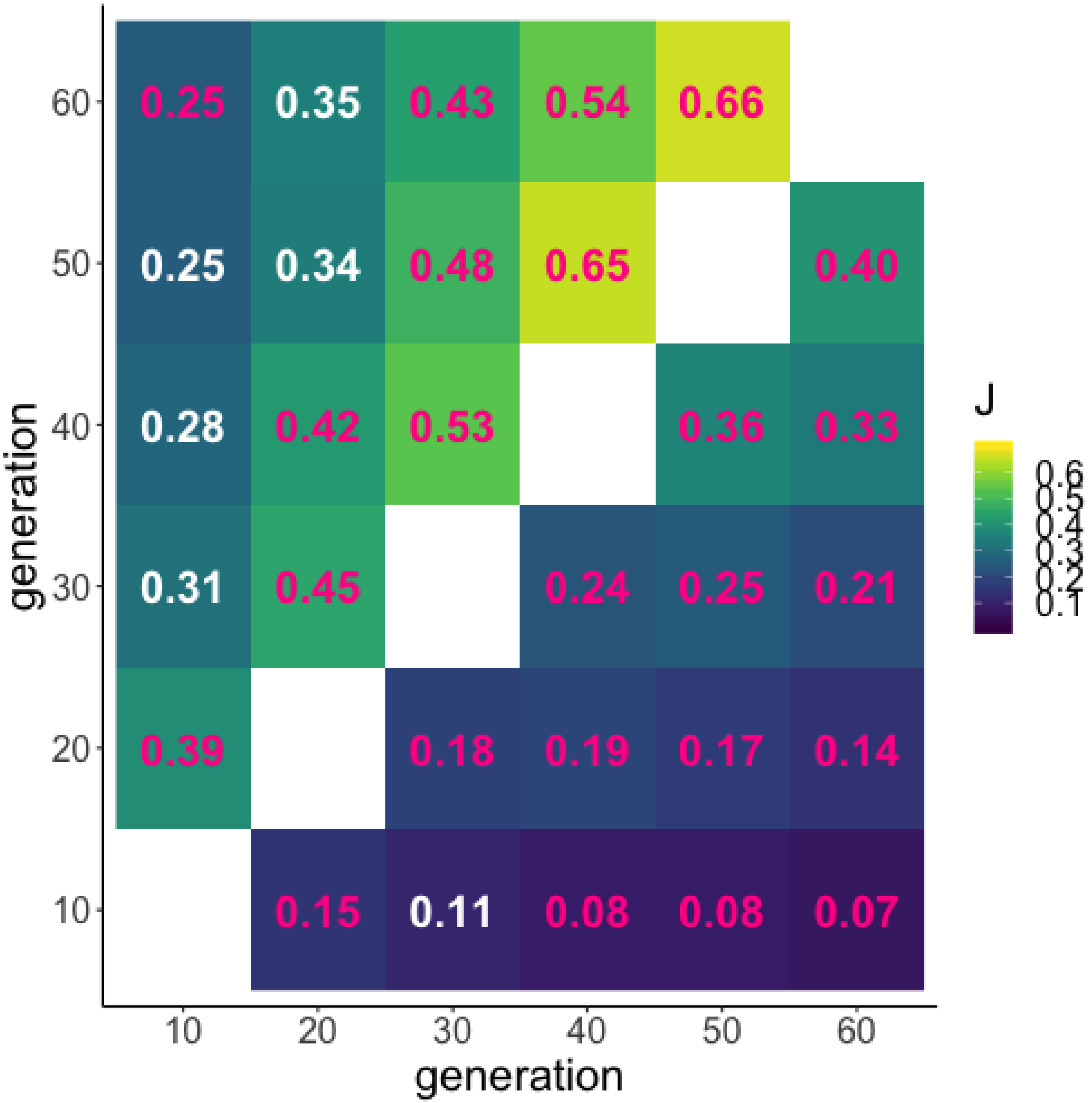

Figure S6: Jaccard index (J) for pairwise comparisons of empirical candidate sets (not filtered for SNPs segregating in all generations and time points). The top triangle shows candidate window sets, the bottom triangle candidate SNP sets. Significant similarities (p<0.05 after multiple testing correction, 10 000 bootstraps) are marked in pink.

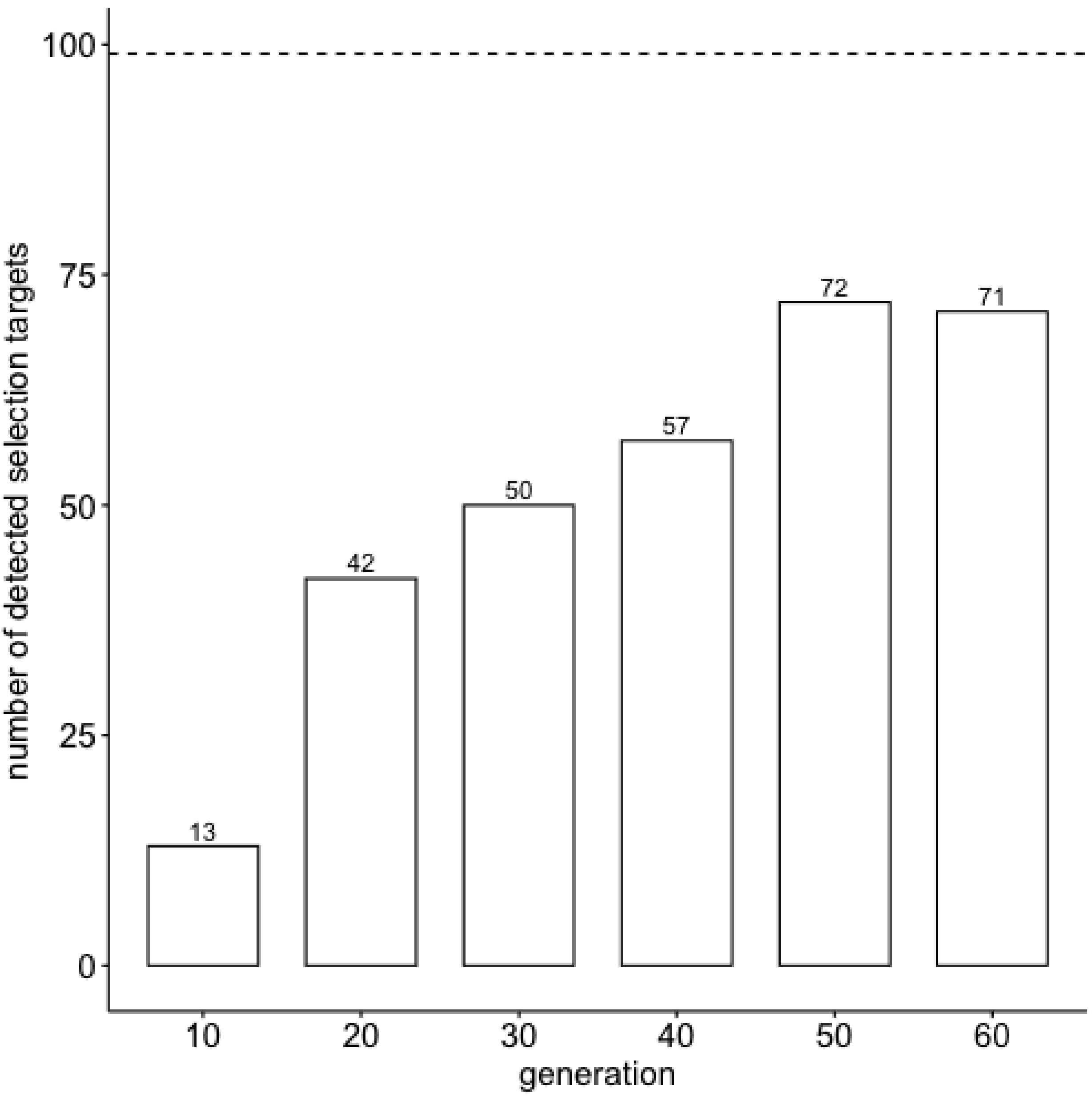

Figure S7: Number of detected causative SNPs for generation 10 to 60. 99 causative SNPs were simulated.

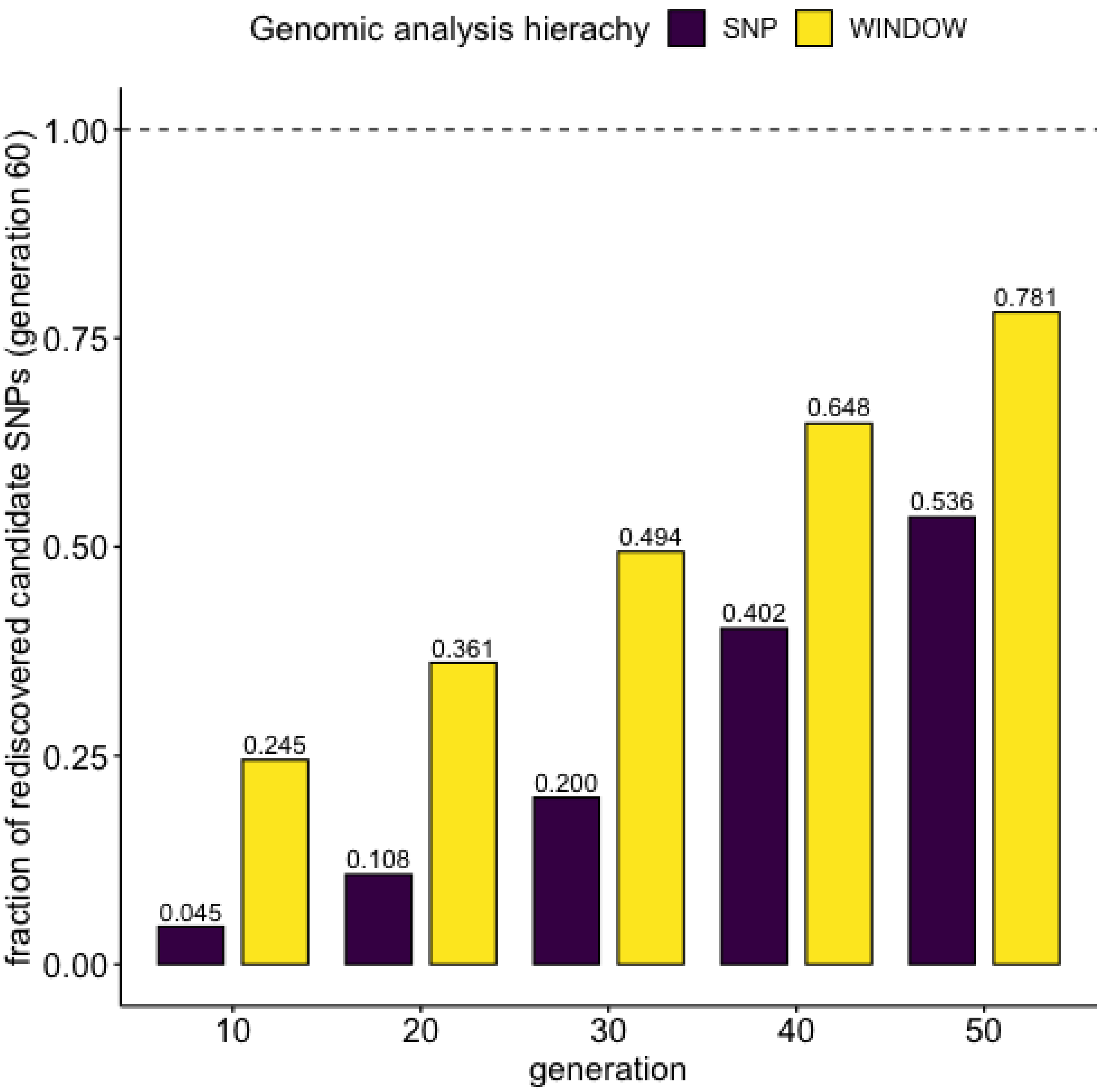

Figure S8: Less than 5% of candidate SNPs in generation 60 are detected consistently at every generation. The bars depict the ratio of candidates at generation 60 (SNPs in purple, windows in yellow) that consistently remain candidates at consecutive earlier time points (e.g. 40.2 % of the candidate SNPs at generation 60 are also candidates in generation 50 and 40). Window-based analyses are more consistent than SNP-based analyses.

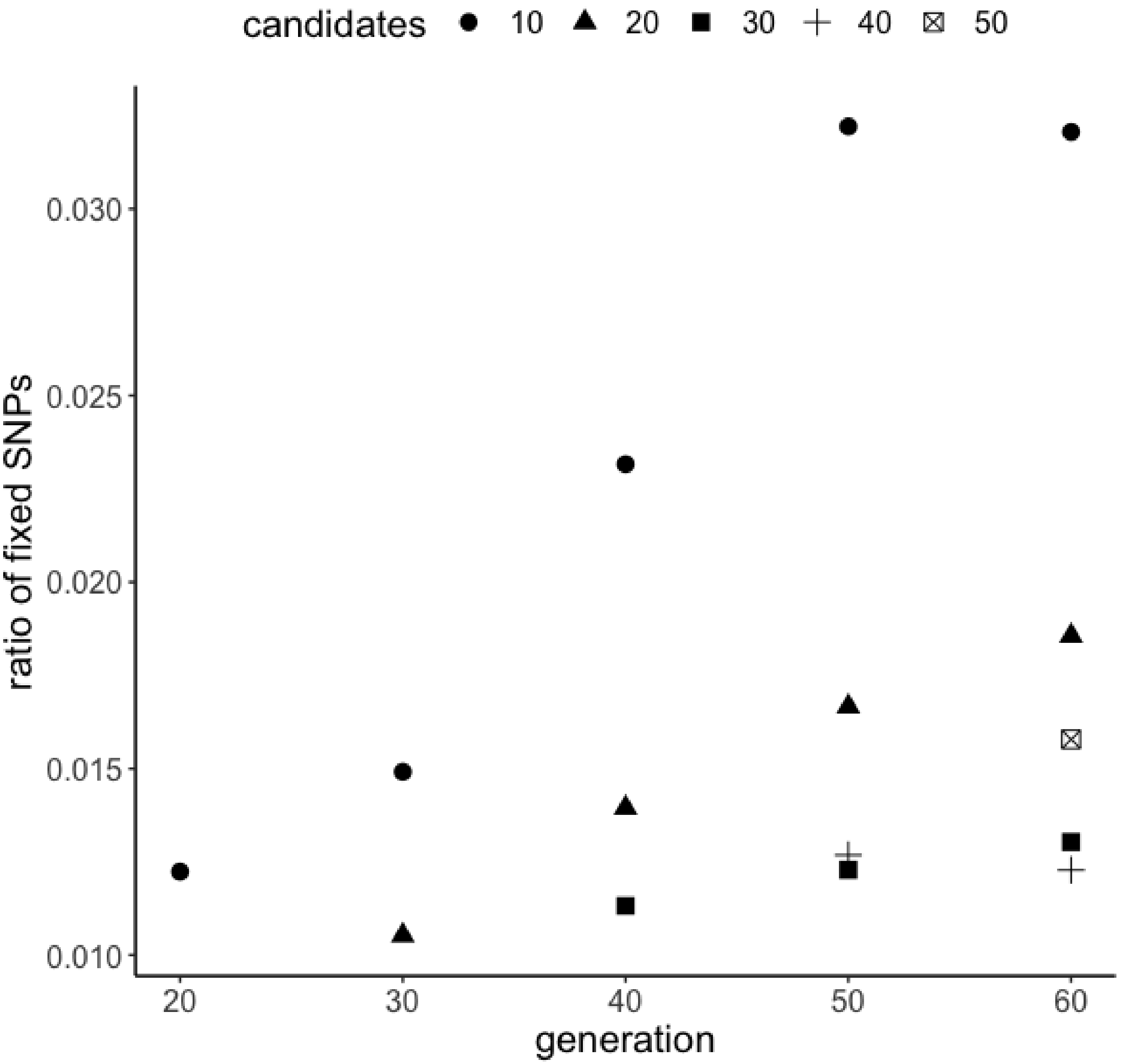

Figure S9: Less than 3.5 % of candidate SNPs in generation 10 are fixed at later generations. The points show the ratio of candidate SNPs that are fixed (frequency ≥ 0.99) in at least 8 out of 10 replicates at later time points.

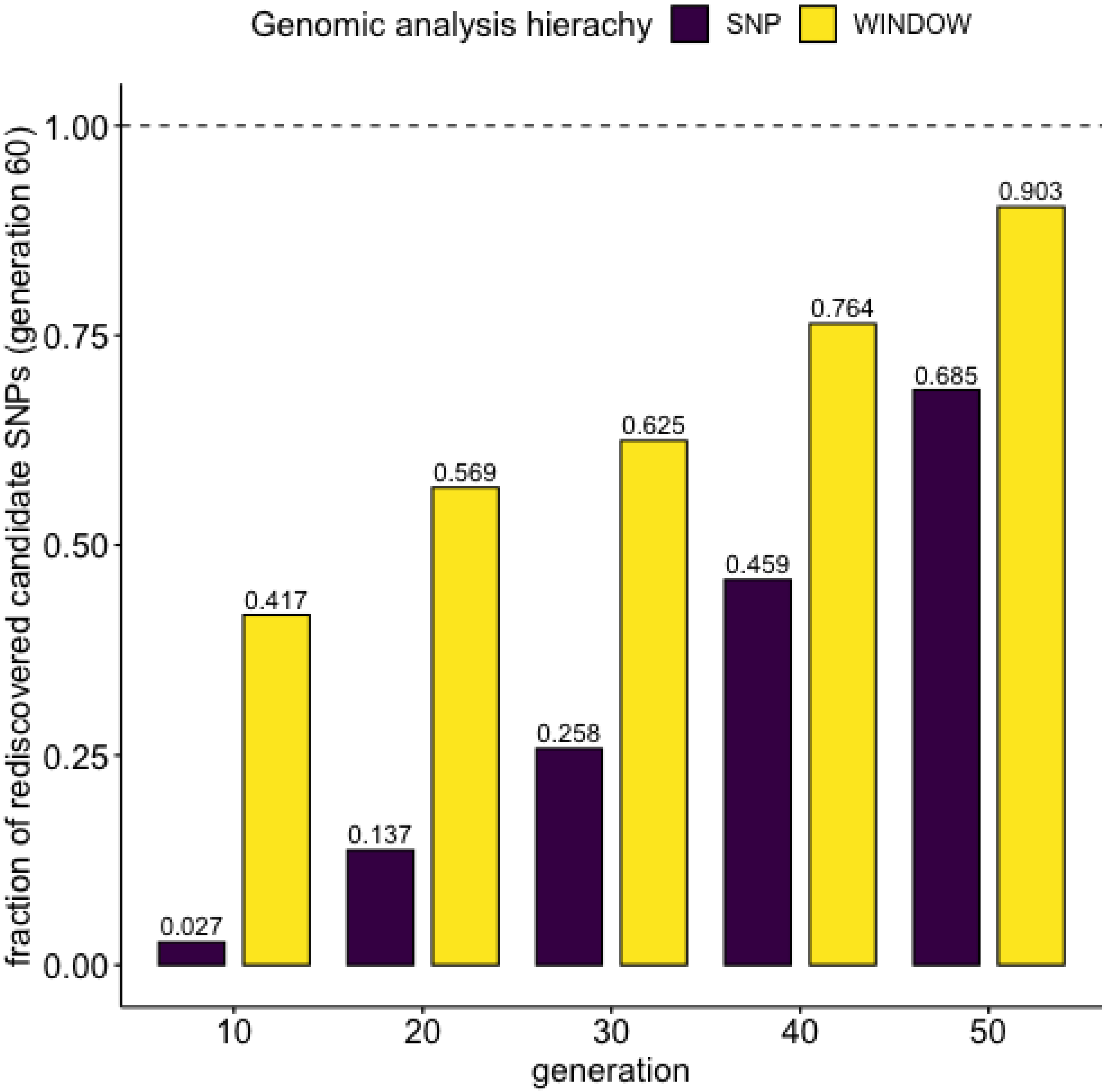

Figure S10: Less than 3% of candidate SNPs in generation 60 are detected consistently at every generation in our simulated Evolve and Resequence experiment. The bars depict the ratio of candidates at generation 60 (SNPs in purple, windows in yellow) that consistently remain candidates at consecutive earlier time points (e.g. 45.9 % of generation 60 candidate SNPs are also candidates in generation 50 and 40). Window-based analyses are more consistent than SNP-based analyses.

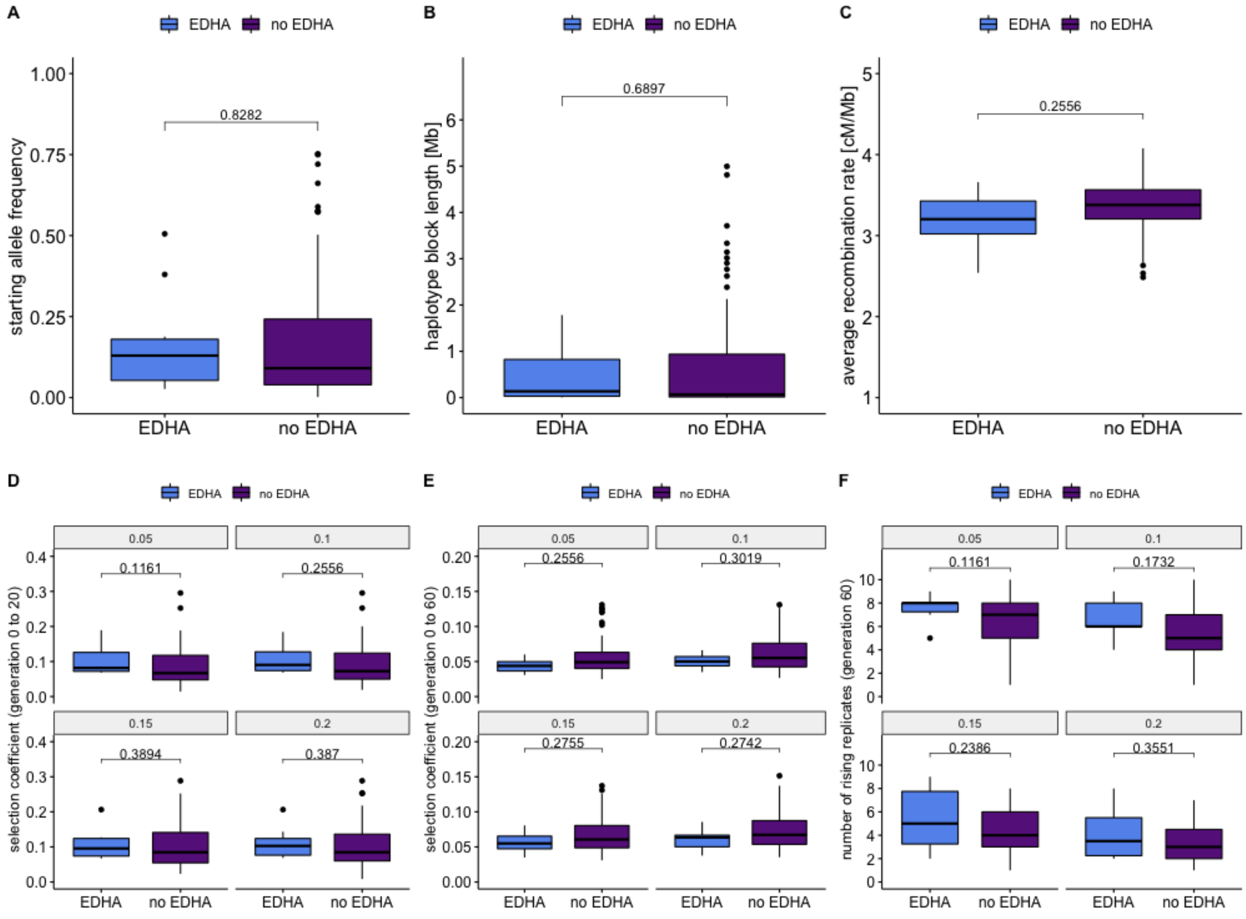

Figure S11: Early detectable haplotype blocks (EDHAs) do not differ in starting allele frequency (**A**), block length (**B**), average recombination rate (**C**), strength of selection (both early (**D**) and late (**E**) in the experiment), or number of rising replicates in generation 60 (**F**) from other haplotype blocks. Values above the boxplots represent the two-tailed Mann-Whitney test *p*-values corrected for multiple testing with the Benjamini-Hochberg procedure.

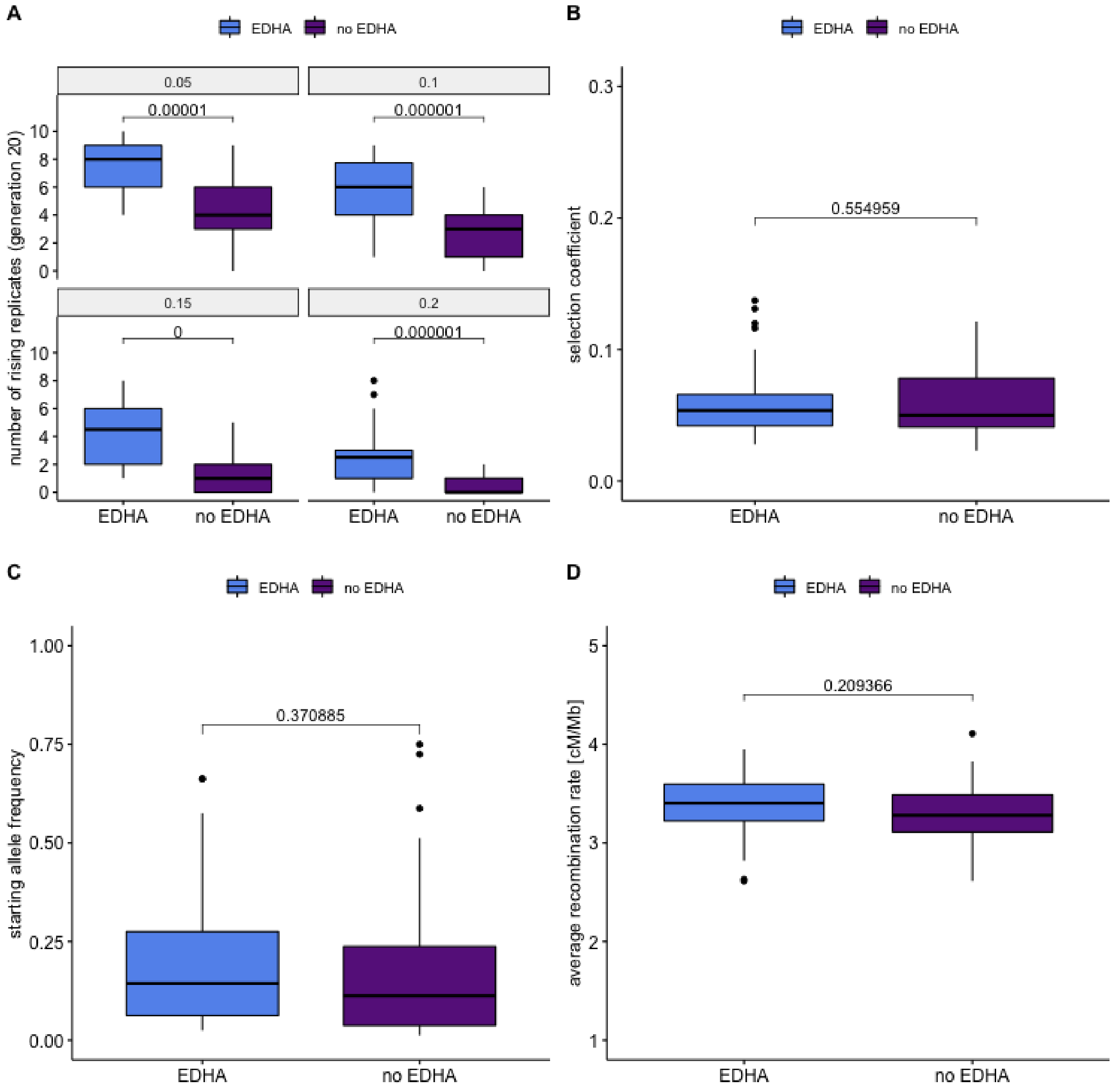

Figure S12: After 20 generations, early detectable targets respond in more replicates than other targets in the simulated Evolve and Resequence experiment – regardless of the chosen allele frequency change cutoff (**A**). Early detectable targets do not differ in selection strength (**B**), starting allele frequency (**C**) or average recombination rate (**D**) from other selected targets. Values above the boxplots represent the two-tailed Mann-Whitney test p-values corrected for multiple testing with the Benjamini-Hochberg procedure.

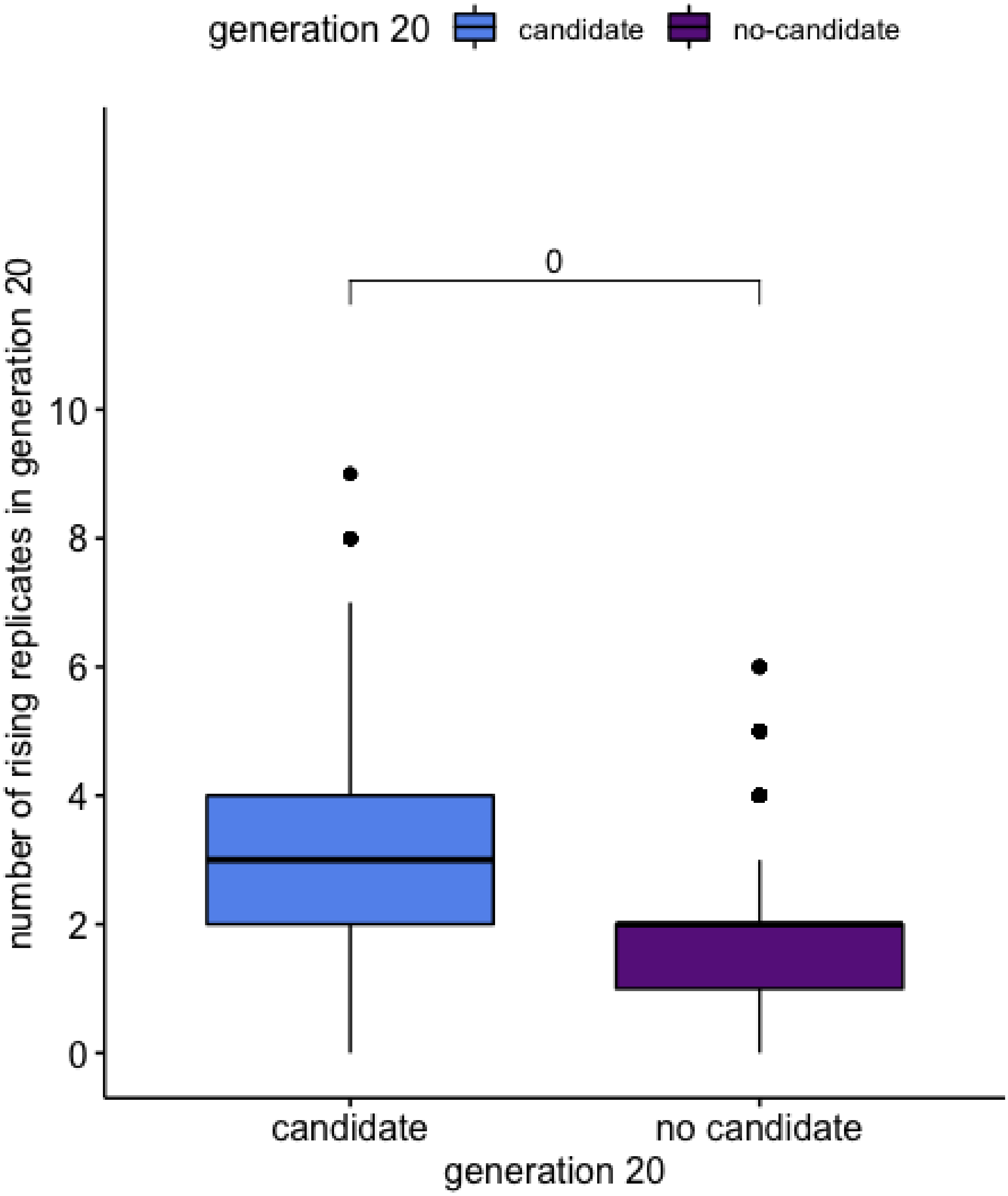

Figure S13: After 20 generations, detectable loci (candidates, FDR≤5%) respond in more replicates (allele frequency change of at least 5%) than not detectable loci, despite having the same starting allele frequency (10%), and selection coefficient (0.05) in computer simulations (100 000 loci simulated). The p-value above the boxplots represents the two-tailed Mann-Whitney test.

Table S1: N_e estimates for the X-chromosome and autosomes averaged across 10 replicates at different time points

generation	X	autosome
10	257	278
20	230	261
30	237	266
40	239	260
50	245	269
60	262	291

Table S2: Replicate-specific autosomal N_e estimates for different time points

generation	R1	R2	R3	R4	R5	R6	R7	R8	R9	R10
10	334	431	333	279	213	251	236	257	262	188
20	309	310	240	208	226	274	221	274	316	235
30	337	304	238	210	234	290	239	243	288	275
40	341	306	238	205	227	287	255	211	270	257
50	356	306	237	235	215	289	281	226	288	257
60	381	336	247	255	246	307	297	244	310	287

Table S3: Replicate-specific X N_e estimates for different time points

generation	R1	R2	R3	R4	R5	R6	R7	R8	R9	R10
10	291	411	279	261	209	228	224	251	211	201
20	181	269	220	226	236	191	255	253	240	230
30	190	281	251	207	237	218	233	239	266	253
40	191	268	265	213	220	230	252	217	286	248
50	203	279	260	231	214	236	256	229	292	250
60	217	280	269	240	208	274	270	241	358	268

Table S4: Fraction of candidate windows at generation 60, that are also candidate windows in all previous generations. a: windows size = 5 000 SNPs. SNPs do not necessarily segregate in all replicates and time points. b: window size =13 025 SNPs (a window with 13 025 SNPs result on average in a window length of 250 kb: that is half the step size in the sliding window approach used by Barghi et al. (2019) to reconstruct selected haplotype blocks) . SNPs do not necessarily segregate in all replicates and time points. c: window size =5 000 SNPs, which segregate in all replicates and time points.

a	b	c
0.24%	0.28%	0.24%

Table S5: Absolute number and fraction of candidate windows at each generation. a: windows size = 5 000 SNPs. SNPs do not necessarily segregate in all replicates and time points. b: window size =13 025 SNPs. SNPs do not necessarily segregate in all replicates and time points. c: window size =5 000 SNPs, which segregate in all replicates and time points.

generation	a	b	c
10	186 (0.18)	78 (0.20)	62 (0.17)
20	224 (0.22)	109 (0.28)	83 (0.23)
30	196 (0.19)	90 (0.23)	72 (0.20)
40	235 (0.23)	111 (0.28)	83 (0.23)
50	225 (0.22)	92 (0.23)	69 (0.19)
60	233 (0.23)	97 (0.25)	74 (0.21)

Table S6: Number of candidate SNPs at each time point

generation	number of candidate SNPs
10	27 290
20	42 736
30	31 002
40	64 974
50	48 344
60	56 166

Table S7: Number of candidate SNPs at each time point in the simulated Evolve and Resequence data.
true positives: SNPs that are candidate SNPs and s>0
false positives: SNPs that are candidate SNPs and s=0

generation	number of candidates SNPs	true positives	false positives
10	35 954	13	35 941
20	108 129	42	108 087
30	106 441	50	106 391
40	142 696	57	142 639
50	177 052	72	176 980
60	194 573	71	194 502

Table S8: Highest -$\log_{10}$(p−value) in haplotype blocks with HADR fluctuation over time. The first row in the table shows the applied 5 % FDR cut-off for autosomes. The second row contains the highest -$\log_{10}$(p−value) observed. Row three to eleven depict the highest -$\log_{10}$(p−values) for different haplotype blocks. We only depict haplotype blocks with pronounced HADR fluctuations at certain time points.

generation	20	30	40	50	60
cut-off	10	14	15	18	19
max	74.06	66.74	78	102.5	112.85
3.8	15.95	14.1	23.37		
3.12	12.09	13.09	22.02		
3.33	18.65	14.69	19.34		
3.24	22.86	12.48	20.87		
3.9	15.30	13.58	24.56		
2.23	19.17	13.55	24.74		
2.7	17.05	18.76	23.00		
3.47			21.44	26.8	30.85
2.15			32.21	32.63	30.03
